# Supplementary material for: The COVID-19 pandemic in Norway and Sweden – threats, trust, and impact on daily life: a comparative survey
Source: BMC Public Health. 2020 Oct 23;20:1597. doi: 10.1186/s12889-020-09615-3 (PMC7582026; doi:10.1186/s12889-020-09615-3)
Supplement: Supplementary file 2 — Additional file 2: Appendix 1b: Questions and responses for surveys performed in Norway (March 20–21) and Sweden (April 10–15), 2020. English version. [file 12889_2020_9615_MOESM2_ESM.docx]

Appendix 1

# Questions and responses for surveys performed in Norway (March 20-21) and Sweden (April 10-15), 2020

**Table 1:** Contacts and experience with health care during the pandemic, number of responses (%)

|  | Norway (%)  Total n=3000 | Sweden (%)  Total n=508 |
| --- | --- | --- |
| Have had contact with health care system | 719 (24) | 415 (82) |
| Did not reach anybody | 65 (9) | 12 (13) |
| Was rejected | 77 (11) | 7 (8) |
| Was advised by health care workers (phone or videoconference) | 428 (60) | 39 (42) |
| Was examined by health care workers | 219 (30) | 43 (46) |
| Got prescription | 113 (16) | 24 (26) |
| Was admitted to hospital or other health care institution | 7 (1) | 3 (3) |

**Table 2:** Translated questions and responses from survey performed in Norway March 20-21, 2020. Response alternatives given on a scale from 1-6, where 1 represents “completely disagree” and 6 represents “completely agree”. Number of responses (%).

| On a scale from 1 to 6, how do you agree with the following statement? | | | | | | | | | | | |
| --- | --- | --- | --- | --- | --- | --- | --- | --- | --- | --- | --- |
|  | *Completely disagree* | |  |  | |  | | |  | *Completely agree* | |
|  | *1* | *2* | | | *3* | | *4* | *5* | | | *6* |
| *Trust* |  |  | | |  | |  |  | | |  |
| I have trust in the government during the coronavirus outbreak | 104 (4) | 233 (8) | | | 528 (18) | | 813 (27) | 825 (28) | | | 497 (17) |
| I have trust in the health authorities during the coronavirus outbreak | 46 (2) | 111 (4) | | | 364 (12) | | 657 (22) | 1,089 (36) | | | 733 (24) |
| I have trust in the hospitals during the coronavirus outbreak | 17 (1) | 38 (1) | | | 154 (5) | | 450 (15) | 1,036 (35) | | | 1,305 (44) |
| I have trust in the municipal health care services during the coronavirus outbreak | 37 (1) | 123 (4) | | | 353 (12) | | 733 (25) | 969 (32) | | | 785 (26) |
| I have received adequate information from the health authorities during the coronavirus outbreak | 40 (1) | 111 (4) | | | 344 (12) | | 691 (23) | 991 (33) | | | 823 (27) |
| I think the government is overreacting | 1,721 (57) | 637 (21) | | | 289 (10) | | 178 (6) | 77 (3) | | | 98 (3) |
| I think the media is exaggerating the severity of the coronavirus situation | 1,048 (35) | 739 (25) | | | 543 (18) | | 374 (13) | 150 (5) | | | 140 (5) |
| *Infection control* |  |  | | |  | |  |  | | |  |
| The virus will die out by closing kindergartens and schools | 438 (15) | 442 (15) | | | 682 (23) | | 704 (24) | 427 (14) | | | 307 (10) |
| We can prevent people from dying by closing kindergartens and schools | 126 (4) | 197 (7) | | | 423 (14) | | 647 (22) | 722 (24) | | | 885 (30) |
| Closing of kindergartens and schools is a good measure of infection control | 98 (3) | 127 (4) | | | 293 (10) | | 504 (17) | 630 (21) | | | 1,348 (45) |
| Closing of kindergartens and schools on March 12 was unnecessary | 1,464 (49) | 498 (17) | | | 295 (10) | | 187 (6) | 139 (5) | | | 417 (14) |
| Countries that have not imposed closing of kindergartens and schools are irresponsible | 193 (6) | 231 (8) | | | 627 (21) | | 591 (20) | 505 (17) | | | 853 (28) |
| Countries that have not imposed closing of kindergartens and schools show lack of solidarity with other countries | 222 (7) | 230 (8) | | | 611 (20) | | 584 (20) | 502 (17) | | | 851 (28) |
| Countries that have not imposed closing of kindergartens and schools are doing the right thing | 1,248 (42) | 710 (24) | | | 752 (25) | | 158 (5) | 56 (2) | | | 76 (3) |
| Closing of borders is sensible | 73 (2) | 90 (3) | | | 214 (7) | | 305 (10) | 556 (19) | | | 1,758 (59) |
| I think it’s unreasonable that the restrictions affect those who are not at an increased risk of severe illness because of the corona virus | 2,004 (67) | 462 (15) | | | 231 (8) | | 147 (5) | 76 (3) | | | 80 (3) |
| I am provoked by the government’s measures with regards to the corona virus | 1,429 (48) | 741 (25) | | | 375 (13) | | 245 (8) | 122 (4) | | | 88 (3) |
| The government has made decisions that lack support in medical evidence | 1,198 (40) | 715 (28) | | | 572 (19) | | 284 (10) | 120 (4) | | | 111 (4) |
| *Getting infected personally* |  |  | | |  | |  |  | | |  |
| I try to avoid getting infected by the corona virus | 18 (1) | 14 (1) | | | 66 (2) | | 142 (5) | 473 (16) | | | 2,287 (76) |
| I am scared of getting infected by the corona virus | 289 (10) | 595 (20) | | | 552 (18) | | 592 (20) | 352 (12) | | | 620 (21) |
| I am scared of infecting others with the corona virus | 60 (2) | 96 (3) | | | 153 (5) | | 376 (13) | 622 (21) | | | 1,693 (56) |
| Getting infected by the corona virus is dangerous | 55 (2) | 213 (7) | | | 835 (28) | | 938 (31) | 463 (15) | | | 496 (17) |
| *Effects of the pandemic* |  |  | | |  | |  |  | | |  |
| I believe the current economic crisis will lead to greater challenges for Norway than the pandemic itself | 202 (7) | 226 (8) | | | 664 (22) | | 557 (19) | 523 (17) | | | 828 (28) |
| It is alright that we currently delay treatments of patients with other illnesses | 254 (9) | 376 (13) | | | 710 (24) | | 704 (24) | 504 (17) | | | 452 (15) |
| I am concerned for my personal economy because of the corona virus pandemic | 622 (21) | 484 (16) | | | 398 (13) | | 428 (14) | 349 (12) | | | 716 (24) |
| *Solidarity and social control* |  |  | | |  | |  |  | | |  |
| I comply with the government’s advice | 7 (0) | 14 (1) | | | 27 (1) | | 109 (4) | 647 (22) | | | 2,196 (73) |
| I feel uneasy if others criticize me for not abiding by the recommendations correctly | 640 (21) | 380 (13) | | | 487 (16) | | 471 (16) | 420 (14) | | | 602 (20) |
| I avoid talking to others that disagree with me on the corona virus situation | 1,242 (41) | 593 (20) | | | 562 (19) | | 291 (10) | 148 (5) | | | 164 (6) |
| I feel that others judge my behaviour in this situation | 1,366 (46) | 558 (19) | | | 289 (10) | | 312 (10) | 240 (8) | | | 235 (8) |
| I feel that others judge my family’s behaviour in this situation | 1,566 (52) | 555 (19) | | | 310 (10) | | 258 (9) | 145 (5) | | | 166 (6) |
| I have opinions on other people’s behaviour in this situation | 157 (5) | 208 (7) | | | 434 (15) | | 639 (21) | 633 (21) | | | 929 (31) |
| I am provoked by people who do not comply with the government’s recommendations | 40 (1) | 53 (2) | | | 112 (4) | | 272 (9) | 572 (19) | | | 1,951 (65) |
| There are people I know that do not comply with the government’s recommendations | 687 (23) | 697 (23) | | | 466 (16) | | 506 (17) | 282 (9) | | | 362 (12) |
| I feel I can’t cough in front of others | 475 (16) | 270 (9) | | | 370 (12) | | 563 (19) | 513 (17) | | | 809 (27) |
| I am not telling others if I have a cold or could be infected | 1,907 (64) | 540 (18) | | | 245 (8) | | 172 (6) | 73 (2) | | | 63 (2) |
| It is shameful to tell others if I am infected | 2,105 (70) | 397 (13) | | | 201 (7) | | 151 (5) | 71 (2) | | | 75 (3) |
| *Changes in everyday life* |  |  | | |  | |  |  | | |  |
| I am depressed | 1,456 (49) | 547 (18) | | | 378 (13) | | 384 (13) | 138 (5) | | | 97 (3) |
| I am apathetic | 1,221 (41) | 566 (19) | | | 426 (14) | | 446 (15) | 213 (7) | | | 128 (4) |
| I am sad | 758 (25) | 482 (16) | | | 536 (18) | | 559 (19) | 325 (11) | | | 335 (11) |
| I feel like my life is put on hold | 360 (12) | 265 (9) | | | 449 (15) | | 612 (20) | 523 (17) | | | 791 (26) |
| I am proud of how I am dealing with this situation | 89 (3) | 148 (5) | | | 574 (19) | | 671 (22) | 669 (22) | | | 849 (28) |
| I feel useful | 363 (12) | 359 (12) | | | 544 (18) | | 523 (17) | 466 (16) | | | 745 (25) |
| I am currently living my life as usual | *381 (13%) svarte Ja*; *2619 (87%) svarte Nei* | | | | | | | | | | |
| I can’t get exercise | 908 (35) | 384 (15) | | | 347 (13) | | 363 (14) | 221 (8) | | | 396 (15) |
| I sit more still than usual | 318 (12) | 229 (9) | | | 266 (10) | | 356 (14) | 383 (15) | | | 1,067 (41) |
| I am more outside than usual | 953 (36) | 441 (17) | | | 414 (16) | | 335 (13) | 194 (7) | | | 282 (11) |
| I eat more than usual | 640 (24) | 405 (16) | | | 425 (16) | | 469 (18) | 264 (10) | | | 416 (16) |
| I drink more alcohol than usual* | 1,248 (74) | 149 (9) | | | 91 (5) | | 98 (6) | 35 (2) | | | 56 (3) |
| My circadian rhythm has changed | 1,258 (48) | 431 (17) | | | 304 (12) | | 281 (11) | 136 (5) | | | 207 (8) |
| I sleep less than usual | 1,215 (47) | 383 (15) | | | 407 (16) | | 237 (9) | 163 (6) | | | 206 (8) |
| I sleep more than usual | 944 (36) | 447 (17) | | | 357 (14) | | 328 (13) | 219 (8) | | | 317 (12) |
| I am not seeing friends | 32 (1) | 32 (1) | | | 86 (3) | | 151 (6) | 481 (18) | | | 1,837 (70) |
| *Conscience* |  |  | | |  | |  |  | | |  |
| I would feel bad if I get quarantined and can’t contribute | 1,195 (40) | 406 (14) | | | 328 (11) | | 318 (11) | 297 (10) | | | 456 (15) |
| I would feel bad to have taken another person’s place in the health care system if I get ill with the corona virus | 1,129 (37) | 442 (15) | | | 388 (13) | | 385 (13) | 293 (10) | | | 363 (12) |
| I would feel bad if I need health care now due to other causes than the corona virus | 1,067 (36) | 423 (14) | | | 348 (12) | | 397 (13) | 324 (11) | | | 441 (15) |
| *Threat* |  |  | | |  | |  |  | | |  |
|  | *Very small* | *Small* | | | *Moderate* | | *Large* | *Very large* | | | *Don’t know* |
| How great of a threat do you feel the corona virus is to your personal health? | 543 (18) | 994 (33) | | | 950 (32) | | 400 (13) | 97 (3) | | | 16 (1) |
| How great of a threat do you feel the corona virus is to the health of others in your family? | 138 (5) | 444 (15) | | | 1,018 (34) | | 840 (28) | 546 (18) | | | 14 (1) |
| How great of a threat do you feel the corona virus is to the public health? | 34 (1) | 267 (9) | | | 1,098 (37) | | 1,172 (39) | 413 (14) | | | 16 (1) |
| How great of a threat do you feel the repercussions of the infection control measures are to Norway? | 17 (1) | 123 (4) | | | 585 (20) | | 1,215 (41) | 921 (31) | | | 139 (5) |

**Footnote:**

*Question added to the survey on March 21, and therefore received only 1,677 responses

**Table 3:** Translated questions and responses from survey performed in Sweden April 10 - 15, 2020. Response alternatives given on a scale from 1-6, where 1 represents “completely disagree” and 6 represents “completely agree”. Number of responses (%).

|  |  |  | On a scale from 1 to 6, how do you agree with the following statement? | | | | | | |
| --- | --- | --- | --- | --- | --- | --- | --- | --- | --- |
|  | | | | *Completely disagree* |  |  |  |  | *Completely agree* |
|  | | | | *1* | *2* | *3* | *4* | *5* | *6* |
| *Trust* | | | |  |  |  |  |  |  |
| I have trust in the government during the coronavirus outbreak | | | | 20 (4) | 20 (4) | 54 (11) | 104 (20) | 122 (24) | 188 (37) |
| I have trust in the health authorities during the coronavirus outbreak | | | | 17 (3) | 14 (3) | 33 (7) | 61 (12) | 129 (25) | 254 (50) |
| I have trust in the hospitals during the coronavirus outbreak | | | | 3 (1) | 9 (2) | 33 (7) | 85 (17) | 155 (31) | 223 (44) |
| I have trust in the municipal health care services during the coronavirus outbreak* | | | | 7 (1) | 31 (6) | 89 (18) | 121 (24) | 122 (24) | 138 (27) |
| I have received adequate information from the health authorities during the coronavirus outbreak | | | | 11 (2) | 12 (2) | 29 (6) | 57 (11) | 130 (26) | 269 (53) |
| I think the government is overreacting | | | | 342 (67) | 102 (20) | 40 (8) | 12 (2) | 8 (2) | 4 (1) |
| I think the media is exaggerating the severity of the coronavirus situation | | | | 186 (37) | 118 (23) | 86 (17) | 64 (13) | 29 (5) | 25 (5) |
| *Measures of disease control* | | | |  |  |  |  |  |  |
| Do you think it’s good that Sweden hasn’t closed preschools and elementary schools?* | | | | 415 (82) answered «Yes»; 44 (9) answered «No»; 48 (9) answered «Don’t know» | | | | | |
| What do you think the effects of closing preschools and elementary schools would have been? | | | | | | | | | |
| The virus would die out* | | | | Yes | | | 61 (12) | | |
| Fewer deaths* | | | | Yes | | | 14 (3) | | |
| A large proportion of the workforce in the health care services would have to stay home with their children - the quality of health care would be substandard* | | | | Yes | | | 435 (86) | | |
| The children’s development and learning would be negatively affected* | | | | Yes | | | 274 (54) | | |
| I am against closing of preschools and elementary schools* | | | | 67 (13) | 26 (5) | 54 (11) | 76 (15) | 74 (15) | 211 (42) |
| Countries that have not imposed closing of kindergartens and schools are irresponsible | | | | 299 (59) | 57 (11) | 36 (7) | 25 (5) | 18 (4) | 73 (14) |
| Countries that have not imposed closing of kindergartens and schools show lack of solidarity with other countries | | | | 346 (68) | 63 (12) | 52 (10) | 18 (4) | 10 (2) | 19 (4) |
| Sweden has done the right thing by not closing kindergartens and schools* | | | | 17 (3) | 18 (4) | 57 (11) | 81 (16) | 89 (18) | 246 (48) |
| It is a good thing that the borders are closed | | | | 29 (6) | 41 (8) | 85 (17) | 71 (14) | 92 (18) | 190 (37) |
| I think it’s unreasonable that the restrictions also affect those who are not at an increased risk of severe illness because of the corona virus | | | | 356 (70) | 66 (13) | 39 (8) | 19 (4) | 11 (2) | 17 (3) |
| I am provoked by the government’s measures with regards to the corona virus | | | | 300 (59) | 111 (22) | 48 (9) | 24 (5) | 13 (3) | 12 (2) |
| There is medical evidence behind the decisions made by the government* | | | | 17 (3) | 25 (5) | 68 (13) | 93 (18) | 121 (24) | 184 (36) |
| *Getting infected personally* | | | |  |  |  |  |  |  |
| I try to avoid getting infected by the corona virus | | | | 4 (1) | 8 (2) | 25 (5) | 71 (14) | 122 (24) | 274 (55) |
| I am scared of getting infected by the corona virus | | | | 58 (11) | 83 (16) | 116 (23) | 88 (17) | 62 (12) | 101 (20) |
| I am scared of infecting others with the corona virus | | | | 15 (3) | 25 (5) | 49 (10) | 66 (13) | 112 (22) | 241 (47) |
| Getting infected by the corona virus is dangerous | | | | 10 (2) | 42 (8) | 132 (26) | 131 (26) | 82 (16) | 111 (22) |
| *Effects of the pandemic* | | | |  |  |  |  |  |  |
| I believe the current economic crisis will lead to greater challenges for Sweden than the pandemic itself | | | | 27 (5) | 32 (6) | 104 (20) | 101 (20) | 108 (21) | 136 (27) |
| It is alright that we currently don’t prioritize treatments of patients with other illnesses | | | | 76 (15) | 71 (14) | 135 (27) | 111 (22) | 51 (10) | 64 (13) |
| I am concerned for my personal economy because of the corona virus pandemic | | | | 98 (19) | 87 (17) | 73 (14) | 90 (18) | 59 (12) | 101 (20) |
| I am concerned that my movements are registered by cell towers | | | | 347 (68) | 64 (13) | 47 (9) | 22 (4) | 12 (2) | 16 (3) |
| *Solidarity and social control* | | | |  |  |  |  |  |  |
| I comply with the government’s advice | | | | 1 (0) | 3 (1) | 13 (3) | 38 (7) | 146 (29) | 307 (60) |
| I feel uneasy if others criticize me for not abiding by the recommendations correctly | | | | 144 (28) | 90 (18) | 101 (20) | 69 (14) | 37 (7) | 67 (13) |
| I avoid talking to others that disagree with me on the corona virus situation | | | | 237 (47) | 105 (21) | 92 (18) | 41 (8) | 17 (3) | 16 (3) |
| I feel that others judge my behaviour with regards to the pandemic | | | | 171 (34) | 89 (18) | 73 (14) | 83 (16) | 49 (10) | 43 (8) |
| I feel that others judge my family’s behaviour regards to the pandemic | | | | 193 (38) | 93 (18) | 80 (16) | 67 (13) | 39 (8) | 36 (7) |
| I have opinions on other people’s behaviour regards to the pandemic | | | | 22 (4) | 50 (10) | 100 (20) | 105 (21) | 104 (21) | 127 (25) |
| I am provoked by people who do not comply with the government’s recommendations | | | | 8 (2) | 24 (5) | 28 (6) | 69 (14) | 112 (22) | 267 (53) |
| I feel it’s okay to criticize the disease control measures that have been taken | | | | 93 (18) | 83 (16) | 132 (26) | 76 (15) | 60 (12) | 64 (13) |
| There are people I know that do not comply with the government’s recommendations | | | | 59 (12) | 117 (23) | 89 (18) | 104 (20) | 68 (13) | 71 (14) |
| I feel I can’t cough in front of others | | | | 43 (8) | 48 (9) | 67 (13) | 117 (23) | 111 (22) | 122 (24) |
| I am not telling others if I have a cold or could be infected | | | | 346 (68) | 90 (18) | 30 (6) | 19 (4) | 9 (2) | 14 (3) |
| It would be shameful to tell others if I am infected | | | | 399 (80) | 58 (11) | 17 (3) | 18 (4) | 8 (2) | 8 (2) |
| *Changes in everyday life* | | | |  |  |  |  |  |  |
| I am depressed | | | | 287 (57) | 89 (18) | 55 (11) | 47 (9) | 19 (4) | 11 (2) |
| I am passive* | | | | 260 (51) | 84 (17) | 68 (13) | 59 (12) | 30 (6) | 7 (1) |
| I feel sad | | | | 143 (28) | 99 (20) | 100 (20) | 71 (14) | 52 (10) | 43 (8) |
| I feel like my life has been put on hold | | | | 45 (9) | 66 (13) | 75 (15) | 99 (20) | 100 (20) | 123 (24) |
| I am proud of how I am dealing with this situation | | | | 17 (3) | 26 (5) | 95 (19) | 106 (21) | 112 (22) | 152 (30) |
| I feel useful | | | | 51 (10) | 48 (9) | 106 (21) | 91 (18) | 94 (19) | 118 (23) |
| I am currently living my life as usual | | | | *101 (19%) answered “Yes”*; *407 (81%) answered “No”* | | | | | |
| I exercise less than usual | | | | 123 (30) | 44 (11) | 37 (9) | 53 (13) | 48 (12) | 102 (25) |
| I sit more still than usual | | | | 110 (27) | 54 (13) | 38 (9) | 60 (15) | 52 (13) | 93 (23) |
| I am more outside than usual | | | | 52 (13) | 38 (9) | 63 (15) | 76 (19) | 66 (16) | 112 (28) |
| I eat more than usual | | | | 140 (35) | 72 (18) | 59 (15) | 64 (16) | 26 (6) | 46 (11) |
| I drink more alcohol than usual | | | | 293 (71) | 48 (12) | 26 (6) | 21 (5) | 8 (2) | 11 (3) |
| My circadian rhythm has changed* | | | | 193 (47) | 46 (11) | 56 (13) | 44 (11) | 34 (8) | 34 (8) |
| I sleep less than usual | | | | 209 (51) | 60 (15) | 69 (17) | 23 (6) | 22 (5) | 24 (6) |
| I sleep more than usual | | | | 161 (40) | 71 (17) | 63 (15) | 49 (12) | 29 (7) | 34 (8) |
| I am not seeing friends | | | | 9 (2) | 21 (5) | 43 (11) | 72 (18) | 97 (24) | 165 (41) |
| *Conscience* | | | |  |  |  |  |  |  |
| I feel bad if I’m quarantined and can’t contribute to society | | | | 247 (49) | 80 (16) | 45 (9) | 51 (10) | 34 (7) | 51 (10) |
| I feel bad for taking another person’s place in the health care system if I need care due to COVID-19 | | | | 230 (45) | 64 (13) | 69 (14) | 51 (10) | 44 (8) | 50 (10) |
| I feel bad if I need health care due to other causes than COVID-19 | | | | 218 (43) | 78 (15) | 72 (14) | 60 (12) | 33 (7) | 47 (9) |
| *Impact* | | | |  |  |  |  |  |  |
|  | | | | *Very small* | *Small* | *Moderate* | *Large* | *Very large* | *Don’t know* |
| How large of a health impact do you feel COVID-19 is to you personally? | | | | 181 (36) | 112 (22) | 101 (20) | 53 (10) | 12 (2) | 49 (10) |
| How large of a health impact do you feel COVID-19 is to others in your family? | | | | 116 (23) | 104 (20) | 143 (28) | 77 (15) | 32 (6) | 36 (7) |
| How large of a health impact do you feel COVID-19 is to the public health? | | | | 9 (2) | 21 (4) | 173 (34) | 208 (41) | 85 (17) | 12 (2) |
| How large are the problems caused by the effects of the disease control measures against the corona virus pandemic in Sweden? | | | | 17 (3) | 45 (9) | 159 (31) | 175 (34) | 107 (21) | 5 (1) |

**Footnote:**

*Questions phrased a little differently than in the Norwegian survey to fit with the Swedish language/the situation in Sweden
