# Supplementary material for: The COVID-19 pandemic in Norway and Sweden – threats, trust, and impact on daily life: a comparative survey
Source: BMC Public Health. 2020 Oct 23;20:1597. doi: 10.1186/s12889-020-09615-3 (PMC7582026; doi:10.1186/s12889-020-09615-3)
Supplement: Supplementary file 4 — Additional file 4: Appendix 2b: Questions and responses from survey performed in Norway, April 4–8, 2020. English version. [file 12889_2020_9615_MOESM3_ESM.docx]

# Appendix 2

## Questions and responses from survey performed in Norway, April 4-8, 2020.

**Table 1**: Demographics of survey respondents, number of responses (%)

| Age |  |
| --- | --- |
| 15-29 | 92 (14,7) |
| 30-49 | 367 (58,4) |
| 50-64 | 125 (19,9) |
| 65 and older | 44 (7,0) |
| Sex |  |
| Women | 437 (69,6) |
| Men | 185 (29,5) |
| Not reported | 6 (1,0) |
| Highest educational level |  |
| Elementary school or upper secondary school | 15 (2.2) |
| High school | 56 (8.9) |
| Higher education <4 years | 167 (26.6) |
| Higher education >4 years | 390 (62.1) |
| Currently attending school or university | 88 (14.0) |
| Number of inhabitants in the residing county |  |
| Rural areas^1^ | 561 (89.3) |
| Urban areas^2^ | 59 (9.1) |
| Unkown county | 10 (1.6) |

^1^ Defined as number of inhabitants less than or equal to 100, 000, ^2^ Defined as number of inhabitants 100,000 and more

**Table 2:** Measures taken to protect themselves and others from COVID-19, number of responses (%)

|  | Protect yourself | Protect others |
| --- | --- | --- |
| Work from home | 406 (64,7) | 405 (64,5) |
| Avoid people that cough | 430 (68,5) | * |
| Cough in elbow hook or disposable handkerchief | * | 533 (84,9) |
| Hand-wash | 619 (98,6) | 620 (98,7) |
| Use glows | * | 182 (29,0) |
| Use face mask | 27 (4,3) | 42 (6,7) |
| Do not meet friends | 510 (81,2) | 523 (83,3) |
| Avoid public transportation | 509 (81,1) | 514 (81,9) |
| Stay home during my spare time | 519 (82,6) | 525 (83,6) |
| No special efforts | 3 (0,5) | 3 (0,5) |

* Did not ask

**Table 3:** Contacts and experience with health care during the pandemic, number of responses (%)

|  | Total n=628 |
| --- | --- |
| Have had contact with health care system | 158 (25.2) |
| Did not reach anybody | 13 (8.2) |
| Was rejected | 9 (5.7) |
| Was advised by health care workers (phone or videoconference) | 90 (57.0) |
| Was examined by health care workers | 55 (34.8) |
| Got prescription | 35 (22.2) |
| Was admitted to hospital or other health care institution | 4 (2.5) |

**Table 4:** Questions and responses from survey performed in Norway April 4-8, 2020. Response alternatives given on a scale from 1-6, where 1 represents “strongly disagree” and 6 represents “strongly agree”. Questions are reproduced in Norwegian, with the exact wording presented to the survey participants. Number of responses (%)

| På en skala fra 1 til 6 - hvor enig er du i følgende påstand? | | | | | | |
| --- | --- | --- | --- | --- | --- | --- |
|  | *Helt uenig* |  |  |  |  | *Helt enig* |
|  | *1* | *2* | *3* | *4* | *5* | *6* |
| *Tillitt* |  |  |  |  |  |  |
| Under utbruddet med koronaviruset har jeg tillit til regjeringen | 11 (1,8) | 48 (7,6) | 80 (12,7) | 164 (26,1) | 190 (30,3) | 135 (21,5) |
| Under utbruddet med koronaviruset har jeg tillit til helsemyndighetene | 4 (0,6) | 21 (3,3) | 53 (8,4) | 117 (18,6) | 225 (35,8) | 208 (33,1) |
| Under utbruddet med koronaviruset har jeg tillit til sykehusene | 4 (0,6) | 8 (1,3) | 29 (4,6) | 72 (11,5) | 218 (34,7) | 297 (47,3) |
| Under utbruddet med koronaviruset har jeg tillit til kommunehelsetjenesten (legevakt, fastlege, sykehjem etc.) | 4 (0,6) | 21 (3,3) | 65 (10,4) | 144 (22,9) | 207 (33,0) | 187 (29,8) |
| Under utbruddet med koronaviruset har jeg fått god informasjon fra helsemyndighetene | 13 (2,1) | 19 (3,0) | 56 (8,9) | 119 (19,0) | 201 (32,0) | 220 (35,0) |
| Jeg synes at myndighetene overreagerer | 288 (45,9) | 165 (26,3) | 60 (9,6) | 58 (9,2) | 25 (4,0) | 32 (5,1) |
| Jeg synes at mediene overdriver alvorligheten i koronavirus-situasjonen | 223 (35,5) | 152 (24,2) | 107 (17,0) | 72 (11,5) | 35 (5,6) | 39 (6,2) |
| *Smittevern* |  |  |  |  |  |  |
| Ved å stenge barnehager og skoler vil viruset dø ut | 150 (23,9) | 114 (18,2) | 139 (22,1) | 122 (19,4) | 69 (11,0) | 34 (5,4) |
| Ved å stenge barnehager og skoler kan vi forhindre at folk dør | 25 (4,0) | 62 (9,9) | 99 (15,8) | 143 (22,8) | 133 (21,2) | 166 (26,4) |
| Å stenge barnehager og skoler er et godt tiltak | 35 (5,6) | 49 (7,8) | 87 (13,9) | 104 (16,6) | 160 (25,5) | 193 (30,7) |
| Land som ikke har innført stengning av skoler og barnehager er uansvarlige | 68 (10,8) | 66 (10,5) | 143 (22,8) | 140 (22,3) | 99 (15,8) | 112 (17,8) |
| Land som ikke har innført stengning av skoler og barnehager er ikke solidariske med andre land | 88 (14,0) | 70 (11,2) | 155 (24,7) | 126 (20,1) | 82 (13,1) | 107 (17,0) |
| Land som ikke har innført stengning av skoler og barnehager gjør det rette | 192 (30,6) | 164 (26,1) | 177 (28,2) | 49 (7,8) | 25 (4,0) | 21 (3,3) |
| Stenging av barnehager og skolen den 12.mars var unødvendig | 285 (45,4) | 124 (19,8) | 66 (10,5) | 28 (4,5) | 47 (7,5) | 78 (12,4) |
| Det er fornuftig å stenge landegrensene | 21 (3,3) | 23 (3,7) | 46 (7,3) | 76 (12,1) | 119 (19,0) | 343 (54,6) |
| Jeg synes det er urimelig at restriksjonene rammer dem som ikke er i risikogruppen for alvorlig sykdom som følge av koronaviruset | 361 (57,5) | 142 (22,6) | 44 (7,0) | 44 (7,0) | 22 (3,5) | 15 (2,4) |
| Jeg er provosert over myndighetenes tiltak for koronaviruset | 316 (50,3) | 172 (27,4) | 70 (11,2) | 38 (6,1) | 17 (2,7) | 15 (2,4) |
| Regjeringen har tatt avgjørelser som det ikke er medisinskfaglig grunnlag for | 188 (29,9) | 142 (22,6) | 119 (19,0) | 76 (12,1) | 54 (8,6) | 49 (7,8) |
| *Egen smitte* |  |  |  |  |  |  |
| Jeg forsøker å unngå å bli smittet av koronaviruset | 4 (0,6) | 6 (1,0) | 18 (2,9) | 41 (6,5) | 132 (21,0) | 427 (68,0) |
| Jeg er redd for å bli smittet av koronaviruset | 50 (8,0) | 152 (24,2) | 130 (20,7) | 127 (20,2) | 79 (12,6) | 90 (14,3) |
| Jeg er redd for å smitte andre med koronaviruset | 13 (2,1) | 27 (4,3) | 59 (9,4) | 90 (14,3) | 148 (23,6) | 291 (46,3) |
| Det er farlig å bli smittet av koronaviruset | 9 (1,4) | 52 (8,3) | 153 (24,4) | 231 (36,8) | 99 (15,8) | 84 (13,4) |
| *Virkning av pandemien* |  |  |  |  |  |  |
| Jeg tror den økonomiske krisen vi ser nå vil føre til større utfordringer for Norge enn selve pandemien | 60 (9,6) | 53 (8,4) | 119 (19,0) | 130 (20,7) | 116 (18,5) | 150 (24,0) |
| Det er greit at vi nå utsetter behandling for pasienter med andre sykdommer | 60 (9,6) | 132 (21,0) | 174 (27,7) | 135 (21,5) | 82 (13,1) | 45 (7,2) |
| Jeg bekymrer meg for min økonomi grunnet coronavirus-pandemien | 203 (32,3) | 119 (19,0) | 84 (13,4) | 83 (13,2) | 54 (8,6) | 85 (13,5) |
| *Solidaritet og sosial kontroll* |  |  |  |  |  |  |
| Jeg følger myndighetenes råd | 3 (0,5) | 5 (0,8) | 8 (1,3) | 29 (4,6) | 185 (29,5) | 398 (63,4) |
| Jeg føler ubehag hvis andre kritiserer meg for ikke å følge anbefalingene godt nok | 108 (17,2) | 63 (10,0) | 123 (19,6) | 123 (19,6) | 117 (18,6) | 94 (15,0) |
| Jeg unngår å snakke med folk som er uenige med meg om koronavirus-situasjonen | 265 (42,2) | 137 (21,8) | 131 (20,9) | 47 (7,5) | 30 (4,8) | 8 (2,9) |
| Jeg opplever at det er greit å kritisere smitteverntiltakene som er iverksatt^1^ | 81 (13,7) | 114 (18,2) | 157 (25,0) | 131 (20,9) | 84 (13,4) | 56 (8,9) |
| Jeg opplever at andre dømmer min atferd i denne situasjonen | 263 (41,9) | 124 (19,8) | 59 (9,4) | 89 (14,2) | 50 (8,0) | 43 (6,9) |
| Jeg opplever at andre dømmer min families atferd i denne situasjonen | 308 (49,0) | 124 (19,8) | 57 (9,1) | 69 (11,0) | 38 (6,1) | 32 (5,1) |
| Jeg har meninger om andres atferd i denne situasjonen | 31 (4,9) | 57 (9,1) | 106 (16,9) | 141 (22,5) | 147 (23,4) | 146 (23,3) |
| Jeg blir provosert av folk som ikke følger myndighetenes anbefalinger | 13 (2,1) | 27 (4,3) | 62 (9,9) | 65 (10,4) | 169 (26,9) | 292 (46,5) |
| Personer jeg kjenner følger ikke myndighetenes råd | 151 (24,0) | 155 (24,7) | 86 (13,7) | 113 (18,0) | 61 (9,7) | 62 (9,9) |
| Jeg føler at jeg ikke kan hoste når andre ser det | 73 (11,6) | 52 (8,3) | 78 (12,4) | 135 (21,5) | 128 (20,4) | 162 (25,8) |
| Jeg forteller det ikke til andre dersom jeg er forkjølet eller kan være smittet | 370 (58,9) | 142 (22,6) | 49 (7,8) | 37 (6,0) | 20 (3,2) | 10 (1,6) |
| Det er skamfullt å fortelle det til andre hvis jeg er smittet | 436 (69,3) | 106 (16,9) | 28 (4,5) | 28 (4,5) | 23 (3,7) | 7 (1,1) |
| *Endring i dagliglivet* |  |  |  |  |  |  |
| Jeg er deprimert | 305 (48,6) | 127 (20,2) | 77 (12,3) | 81 (12,9) | 26 (4,1) | 12 (1,9) |
| Jeg er tiltaksløs | 231 (36,8) | 134 (21,3) | 91 (14,5) | 105 (16,7) | 45 (7,2) | 22 (3,5) |
| Jeg er lei meg | 183 (29,1) | 138 (22,0) | 87 (13,9) | 115 (18,3) | 69 (11,0) | 36 (5,7) |
| Jeg føler at livet mitt er satt på vent | 71 (11,3) | 77 (12,3) | 92 (14,7) | 142 (22,6) | 109 (17,4) | 137 (21,8) |
| Jeg er stolt av hvordan jeg forholder meg til denne situasjonen | 28 (4,5) | 29 (4,6) | 130 (20,1) | 159 (25,3) | 149 (23,7) | 133 (21,2) |
| Jeg føler meg nyttig | 61 (9,7) | 66 (10,5) | 114 (18,2) | 130 (20,7) | 114 (18,2) | 143 (22,8) |
| Jeg lever mitt liv som vanlig | *80 (12,7%) svarte Ja*; *548 (87,3%) svarte Nei* | | | | | |
| Jeg får ikke trent | 209 (38,1) | 94 (17,2) | 68 (12,4) | 65 (11,9) | 52 (9,5) | 60 (11,0) |
| Jeg sitter mer stille enn vanlig | 90 (16,4) | 55 (10,0) | 54 (9,9) | 85 (15,5) | 75 (13,7) | 189 (34,5) |
| Jeg er mer ute enn vanlig | 139 (25,4) | 75 (13,7) | 104 (19,0) | 71 (13,0) | 54 (9,9) | 105 (19,2) |
| Jeg spiser mer enn vanlig | 106 (19,3) | 101 (18,4) | 84 (15,3) | 99 (18,1) | 67 (12,2) | 91 (16,6) |
| Jeg drikker mer alkohol enn jeg vanligvis gjør | 311 (60,4) | 65 (11,9) | 43 (7,9) | 46 (8,4) | 26 (4,7) | 37 (6,8) |
| Søvnrytmen min har endret seg | 183 (33,4) | 74 (13,5) | 58 (10,6) | 100 (18,3) | 51 (9,3) | 82 (15,0) |
| Jeg sover mindre enn vanlig | 252 (46,0) | 79 (14,4) | 91 (16,6) | 59 (10,8) | 25 (4,6) | 42 (7,7) |
| Jeg sover mer enn vanlig | 174 (31,8) | 90 (16,4) | 81 (14,8) | 79 (14,4) | 55 (10,0) | 69 (12,6) |
| Jeg treffer ikke venner | 8 (1,5) | 9 (1,6) | 27 (4,9) | 50 (9,1) | 118 (21,5) | 336 (61,3) |
| Har din arbeidssituasjon endret seg grunnet koronaviruspandemien | *260 (41,4%) svarte Ja;368 (58,6%) svarte Nei* | | | | | |
| Jeg har blitt arbeidsledig | Ja | | | 8 (3,1%) | | |
| Jeg har blitt permittert | Ja | | | 44 (16,9%) | | |
| Annet | Ja | | | 208 (80%) | | |
| *Samvittighet* |  |  |  |  |  |  |
| Jeg får dårlig samvittighet hvis jeg havner i karantene og ikke kan bidra | 264 (42,0) | 108 (17,2) | 65 (10,4) | 63 (10,0) | 53 (8,4) | 75 (11,9) |
| Jeg får dårlig samvittighet over å ha tatt andres plass i helsevesenet hvis jeg blir syk av koronaviruset | 270 (43,0) | 108 (17,2) | 75 (11,9) | 72 (11,5) | 58 (9,4) | 45 (7,2) |
| Jeg får dårlig samvittighet hvis jeg trenger helsehjelp nå av andre årsaker enn koronaviruset | 254 (40,5) | 102 (16,2) | 86 (13,7) | 74 (11,8) | 63 (10,0) | 49 (7,8) |
| *Trussel* |  |  |  |  |  |  |
|  | *Veldig liten* | *Liten* | *Moderat* | *Stor* | *Veldig stor* | *Vet ikke* |
| Hvor stor helsetrussel opplever du at koronaviruset utgjør for deg personlig? | 128 (20,4) | 242 (38,5) | 191 (30,4) | 53 (8,4) | 10 (1,6) | 4 (0,6) |
| Hvor stor helsetrussel opplever du at koronaviruset utgjør for andre i din familie? | 29 (4,6) | 118 (18,8) | 238 (37,9) | 152 (24,2) | 86 (13,7) | 5 (0,8) |
| Hvor stor helsetrussel opplever du at koronaviruset utgjør for befolkningen? | 11 (1,8) | 84 (13,4) | 264 (42,0) | 219 (34,9) | 48 (7,6) | 2 (0,3) |
| Hvor stor trussel er ringvirkningene av tiltakene mot koronavirus-pandemien for Norge? | 4 (0,6) | 26 (4,1) | 138 (22,0) | 281 (44,8) | 161 (25,6) | 18 (2,9) |
